# Evolution of airblast induced by roof collapse based on LBM-DEM

**DOI:** 10.1038/s41598-025-95805-1

**Published:** 2025-04-03

**Authors:** Mingtao Jia, Rongtao Yang, Shaodong Li, Xiaoqiang Guo, Liguan Wang

**Affiliations:** 1 School of Resources and Safety Engineering, Central South University, Hunan, 410083 Changsha People’s Republic of China; 2 Engineering Research Center of Ministry of Education for Carbon Emission Reduction in Metal Resource Exploitation and Utilization, Changsha, 410083 People’s Republic of China; 3 Zijin Mining Group Co., Ltd, Fujian, 362400 Xiamen People’s Republic of China

**Keywords:** Block caving, Airblast, Hazard assessment and prevention, LBM-DEM, Key parameters influencing airblast, Multivariate airblast model, Solid Earth sciences, Engineering

## Abstract

Airblast is a common safety hazard in block caving. It occurs when the roof of a goaf collapses suddenly over a large area, causing rapid compression and release of air, resulting in high-speed airflow that can cause injury and equipment damage. To effectively assess and prevent airblast hazards, it is necessary to study the catastrophic behavior of airblast parameters. The air gap, caving scale, and muckpile height are airblast key parameters. This research employs the LBM-DEM (Coupled Lattice Boltzmann Method and Discrete Element Method) to model the airblast formation process. Combined simulation experiments were conducted to examine the effects of air gap, muckpile height, and caving scale, with sensitivity analysis performed to determine airblast response. We propose a multivariate regression model that, using three arguments, expresses the maximum airblast velocity at the drawpoints. Results show that maximum air velocity during roof collapse is positively correlated with air gap and caving scale, and negatively correlated with muckpile height. At a caving scale of 10^3^ m^3^, the air gap is the most critical parameter affecting maximum air velocity. Moreover, the interactions among these parameters exhibit distinct coupling characteristics. The findings provide a theoretical basis and reference for assessing and preventing airblast hazards.

## Introduction

Recently, newly discovered underground mines in China are often characterized by deep burial and low-grade ore. To efficiently, economically, and safely extract these large-scale, low-grade deposits, block caving is considered a suitable mining method^1,2^. However, during block caving, improper draw control can create a significant void between the caved roof and the muckpile. Before the orebody breaks through to the surface, this situation resembles sublevel caving in an open stope. Once the overlying rock collapses on a large scale, the air in the void is compressed, creating a strong airblast that can harm underground workers and damage equipment^3–7^. Several severe airblast incidents have occurred worldwide. For example, the 1999 Northparkes Mine airblast in Australia resulted in the death of 4 miners and injuries to several others^8^. In China, the 1993 Zaozhuang gypsum mine and the 2006 Shimen Tiande gypsum mine airblast caused over ten and nine deaths, respectively, with airblast speeds reaching 274 m/s in the latter incident^9^.

Given the significant dangers of airblast^10–12^, many scholars have used various models and methods to study the mechanisms and influencing factors of airblast. Zhong et al.^13^ found that the maximum air velocity is positively correlated with the projected area of the collapsed body and air gap, and negatively correlated with the stope area and air flow distance. Wu et al.^14^ studied the airblast attenuation effect of the muckpile through shock tube experiments. Oh et al.^15^ using PFC, studied the effects of muckpile height, muckpile particle size, and porosity on airblast, and found that muckpile porosity is the most effective parameter influencing airblast. Cao et al.^16^ and Zhang^17^ comprehensively evaluated the safe height of the muckpile, Sergio Palma et al.^18^ studied the effects of muckpile permeability and air gap on airblast, Martín Rojas et al.^19^ investigated the effects of air gap and muckpile particle size on airblast. However, most studies focus primarily on muckpile and air gap, with an emphasis on single-factor impact analysis. Although most studies^13,14,16,17,20^ are based on specific mining conditions, their conclusions may have limited applicability in different mining environments, potentially leading to inconsistencies when applied under varying conditions. Therefore, some studies have attempted to enhance the generalizability of their findings by developing multi-variable models^18,19^. However, the parameters used in these models remain insufficiently comprehensive, limiting their applicability in a broader range of mining environments.

This study refers to the natural caving process in mines, utilizing MechSys^21^, and the lattice Boltzmann-discrete element method. It takes caving scale, air gap, and muckpile height as fundamental factors, designing a multi-factor numerical analysis plan to explore and reveal the response mechanisms of airblast disaster factors during caving mining. The numerical model employed in this research was created by Galindo-Torres^22^, with validation and specifics available in the cited literature^22–24^.

## Methodology

### LBM

LBM is a numerical simulation method based on microscopic particle dynamics, originating from the lattice gas automaton (LGA). LBM discretizes space using a symmetric grid, representing the fluid system as a set of fluid particles located in cubic cells of a regular Cartesian grid^25^. The lattice spacing is $\delta x$.The particle distribution function at a lattice node—${f}_{i}(x,t)$ is defined to represent the number of fluid particles moving in direction i at node *x *at time* t*. The LBM evolution equation:

1 $${f}_{i}\left(x+{e}_{i}{\delta }_{t},t+{\delta }_{t}\right)-{f}_{i}(x,t)=-\frac{1}{\tau }\left[{f}_{i}(x,t)-{f}_{i}^{eq}(x,t)\right]$$

where: ${\delta }_{t}$ denotes the time step;${e}_{i}{\delta }_{t}$ represents the lattice length; $\tau$ represents the relaxation time (rate at which the particle distribution function approaches the equilibrium distribution function); $x$ represents position; ${e}_{i}$ denotes the lattice velocity; $i$ denotes the velocity direction; ${f}_{i}^{eq}$ is the equilibrium distribution function.

LBM commonly uses the discretized Maxwell distribution as the equilibrium distribution^26,27^. The equilibrium distribution function is given as follows:

2 $${f}_{i}^{eq}={\omega }_{i}\rho \left(1+3\frac{{\overrightarrow{\varvec{e}}}_{i}\cdot \overrightarrow{u}}{{C}^{2}}+\frac{9{\left(\overrightarrow{{\varvec{e}}_{i}}\cdot \overrightarrow{u}\right)}^{2}}{2{C}^{4}}-\frac{3{u}^{2}}{2{C}^{2}}\right)$$

where ${w}_{i}$ is the weight associated with lattice velocity ${c}_{i}$, $u$ represents fluid velocity. $C=\frac{{\delta }_{x}}{{\delta }_{t}}$, $u$ represents fluid velocity, $\rho$ represents fluid density.

3 $$\rho = \sum _{i=0}^{18}{f}_{i}$$

4 $$u=\frac{{\sum }_{i=0}^{18}{\varvec{c}}_{i}{f}_{i}}{\rho }$$

LBM can recover the nonlinear macroscopic Navier–Stokes equations with second-order accuracy. Therefore, the relationship between the dynamic fluid viscosity, ${ \upsilon }_{f}$, and lattice units can be derived from the LBM evolution equation as:

5 $${v}_{f}={c}_{s}^{2}\left(\tau -\frac{1}{2}\right)\frac{{\delta }_{x}^{2}}{{\delta }_{t}}$$

where ${c}_{s}$ denotes the speed of sound, given by $\frac{C}{\sqrt{3}}$.

Under adiabatic conditions, the equation of state for pressure can be directly obtained from density $\rho$, as shown below:

6 $$p={c}_{s}^{2}{\rho }_{f}$$

### DEM

DEM was first introduced and developed by Cundall et al.^28,29^ in the 1970s and has since been widely used in simulations of discrete systems such as rock mechanics and granular flows. This study uses DEM with the soft-sphere model to simulate interactions between particles. The motion of DEM particles is calculated using Newton’s second law and the Verlet al.gorithm^30^, the equations as follows:

7 $${m}_{i}{a}_{i}={m}_{i}g+{F}_{i}^{c}+{F}_{i}^{f}$$

8 $${I}_{i}\frac{d}{dt}{w}_{i}={T}_{i}^{c}+{T}_{i}^{f}$$

where, ${m}_{i}$ is the mass of the particle,${ a}_{i}$ is the acceleration,${I}_{i}$ is the moment of inertia, ${w}_{i}$ is the angular velocity, $g$ is the gravitational acceleration, ${F}_{i}^{f}$ and ${T}_{i}^{f}$ are the force and torque exerted by the fluid on the particles,${F}_{i}^{c}$ and ${T}_{i}^{c}$ are the force and torque generated by particle collisions.

### LBM-DEM

To accurately handle the interaction between fluid and solid, this paper employs the immersed moving boundary method^31^ to achieve LBM-DEM coupling.

9 $${f}_{i}\left(\overrightarrow{x}+\overrightarrow{{e}_{i}}{\delta }_{t},t+{\delta }_{t}\right)={f}_{i}(\overrightarrow{x},t) +\left(1-{B}_{n}\right)\left(\frac{1}{\tau }\left({f}_{i}^{eq}-{f}_{i}\right)\right)+{B}_{n}{\varOmega }_{i}^{S}$$

where ${B}_{n}$ represents the moving boundary-related function; ${\varOmega }_{i}^{S}$ denotes the additional collision term. The force exerted by the fluid on DEM particles is calculated as follows:

10 $$\overrightarrow{{F}_{i}^{f}}=\frac{{\delta }_{x}^{3}}{{\delta }_{t}}{\sum }_{n}{B}_{n}\left(\sum _{i}{\varOmega }_{i}^{s}{\overrightarrow{e}}_{i}\right)$$

The torque exerted by LBM on DEM particles:

11 $${T}_{i}^{f}=\frac{{\delta }_{x}^{3}}{{\delta }_{t}}\sum _{n}\left[\left({\overrightarrow{x}}_{n}-{\overrightarrow{x}}_{CM}\right)\times {B}_{n}\left(\sum _{i}{\varOmega }_{i}^{s}{\overrightarrow{e}}_{i}\right)\right]$$

where ${\overrightarrow{x}}_{CM}$ is the particle’s center of mass, and ${\overrightarrow{x}}_{n}$ is the coordinate of the n-th lattice cell.

### Model parameters

**Fig. 1:**
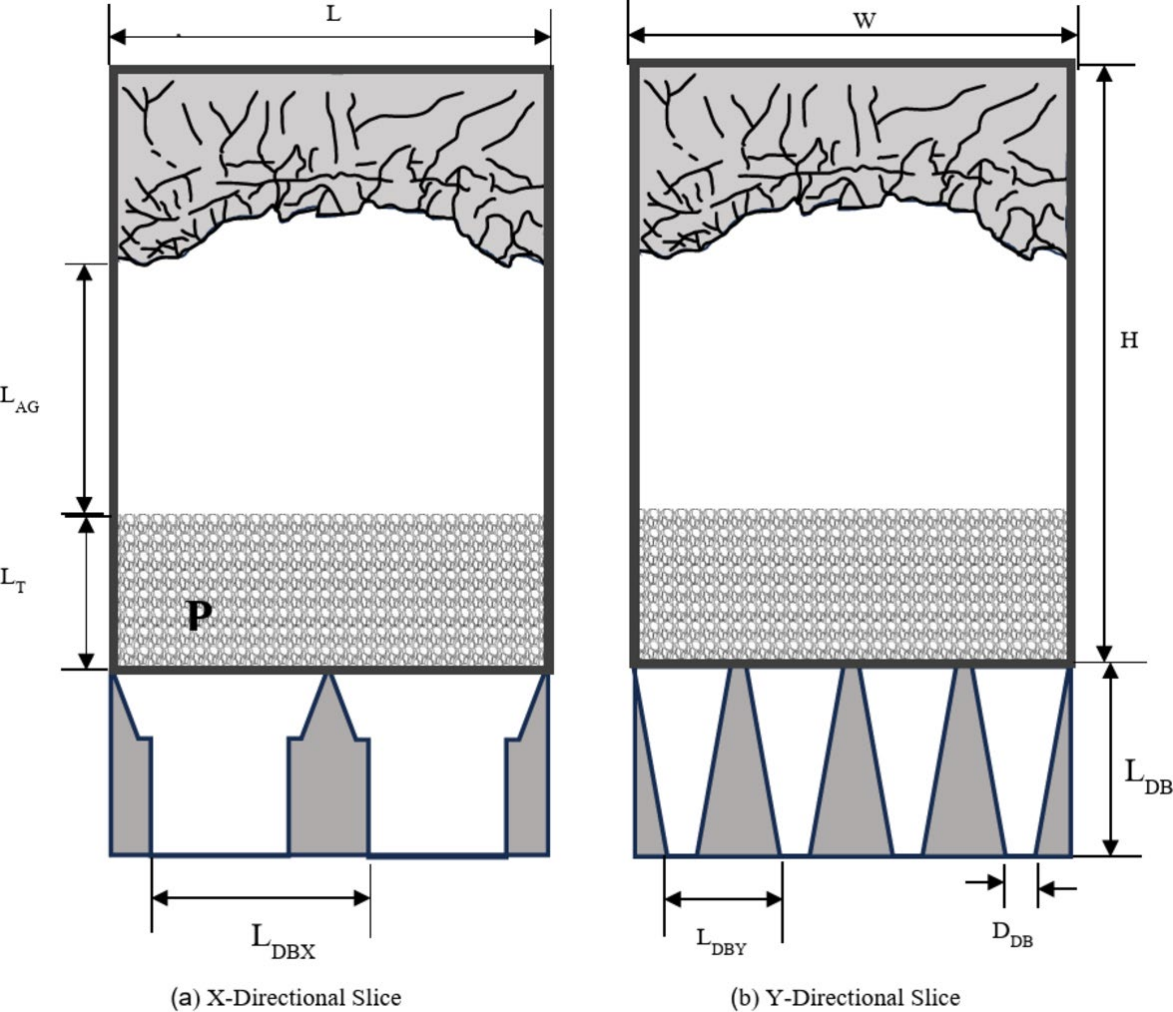
Simplified model.

In this section, the geometric and physical parameters of the numerical simulation model are explained in detail. The simulated model is designed with a rectangular void space measuring 200 × 102 × 105 m, which is connected at the base to 16 gathering hoppers, with density (pressure) boundary conditions^32^ applied at the bottom outlets. The cushion layer utilizes a uniform porous medium with a porosity of 0.3, as illustrated in Fig. 1. For this simulation, only the air gap, muckpile height, and caving scale are varied, with all other parameters kept constant. The specific meanings of the geometric parameters are shown in Fig. 1, with their values summarized in Table 1, the drawbell parameters were derived from real model data from a copper mine in Serbia. Meanwhile, the values for muckpile height, air gap, and caving scale were determined based on studies of airblast in natural caving method, relevant technical regulations, and actual mining disaster case data^8,15,18,19,33–35^. The height, width, and length of the caved void were reasonably designed based on the selected parameters to ensure the engineering applicability of the model. The physical parameter values are summarized in Table 2, with porosity values referenced from^15^, and air viscosity, normal stiffness, and tangential stiffness values referenced from Sergio Palma’s work^18^.

**Table 1:** Geometric parameters.

Parameters	Value
Stope height, H (m)	200
Stope width, W (m)	102
Stope length, L (m)	105
Air gap, L_AG_ (m)	[5, 15, 25, 35]
Muckpile height, L_T_ (m)	[20, 40, 60, 80]
Caving scale, V_R_ (km^3^)	[160, 192, 224, 256]
Drawbell height, L_DB_ (m)	30
Drawbell bottom width, D_DP_ (m)	4.5
Drawbell lateral spacing, L_DBX_ (m)	35
Drawbell horizontal spacing, L_DBY_ (m)	17

**Table 2:** Physical parameters.

Parameters	Value
Porosity muckpile, P	0.3
Kinematic viscosity air, ${\nu }_{f}$ (m^2^/s)	2.5e−05
Normal stiffness rocks, ${K}_{n}$ (N/m)	1e10
Tangential stiffness rocks, ${K}_{t}$ (N/m)	1e10

## Results and analysis

This section assesses the influence of air gap, muckpile height, and caving scale on airblast based on numerical simulation results. To quantify airblast hazards, a criterion is necessary. The maximum air velocity at the drawpoint during rockfall is used to determine the airblast hazard, with this value serving as the standard for assessing disaster severity.

Given the lack of definitive threshold values for equipment damage, human injury, and fatal wind speeds, this study references the findings of Xing^36^ and McIlveen^37^ and sets the critical wind speeds for equipment damage, human injury, and fatalities at 85.3 m/s, 19.7 m/s, and 58.5 m/s, respectively.

### Effect of air gap

**Fig. 2:**
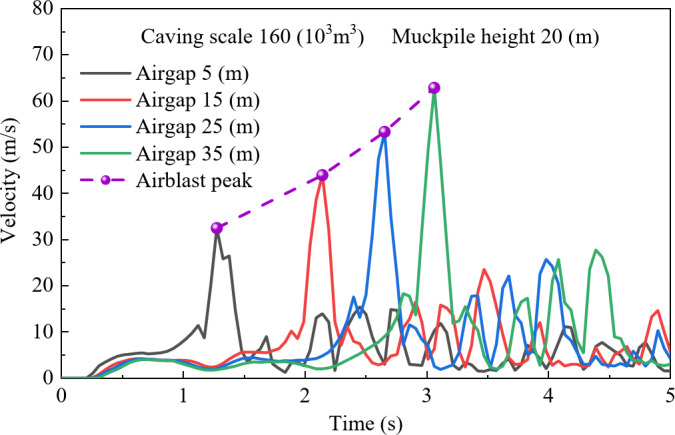
Air velocity evolution curves with different airgap.

To investigate the effect of air gap on airblast velocity, the study analyzed the velocity distribution for a caving scale of 160 × 10^3^ m^3^ and a muckpile height of 20 m under four different air gaps 5 m, 15 m, 25 m, and 35 m. As shown in Fig. 2, during the initial stage of rock caving, the airblast velocity increases over time, surging sharply when the rock reaches the muckpile, then rapidly dropping and fluctuating. This fluctuation is caused by the compression of air by falling rock, leading to a rapid increase in pressure, followed by a decline as the gas diffuses. Meanwhile, as the air gap increases, the general trend of the airblast velocity evolution curve remains similar, but the peak airblast velocity shows a positive correlation with air gap, reaching 32.479 m/s, 43.944 m/s, 53.31 m/s, and 62.837 m/s for air gaps of 5 m, 15 m, 25 m, and 35 m, respectively. This phenomenon can be attributed to the increased kinetic energy of the falling rock as the air gap increases. Furthermore, a delay in the peak velocity is observed at different air gaps, due to the extended time required for rockfall as the air gap increases.

**Fig. 3:**
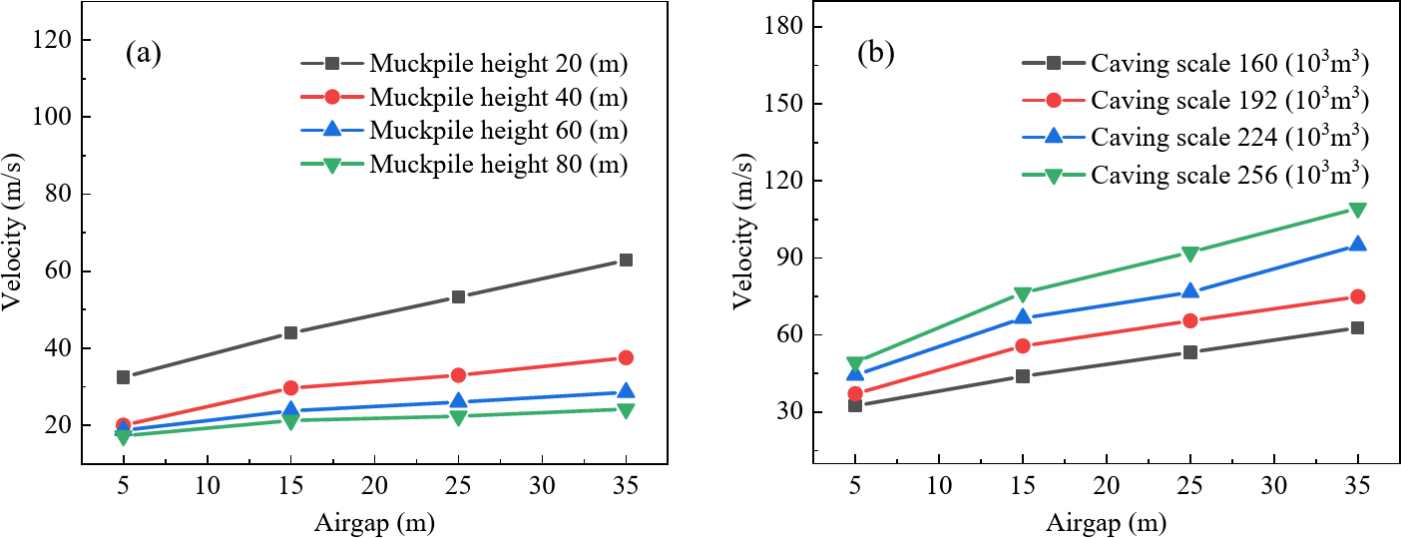
Relationship between air gap and maximum air velocity; (**a**) Effect of muckpile height on the relationship between air gap and maximum air velocity; (**b**) Effect of caving scale on the relationship between air gap and maximum air velocity.

To clarify whether the influence of air gap on the maximum air velocity at the drawpoint is affected by caving scale and muckpile height, the impact of air gap on maximum air velocity was analyzed for four muckpile heights (20 m, 40 m, 60 m, and 80 m) and four caving scales (160 × 10^3^ m^3^, 192 × 10^3^ m^3^, 224 × 10^3^ m^3^, and 256 × 10^3^ m^3^). Figure 3a shows that the maximum air velocity at the drawpoint is positively correlated with air gap, and as muckpile height increases, the velocity increment decreases. This is because the increased muckpile height results in a longer buffering time for the airblast, which in turn reduces the velocity. With muckpile heights of 20 m, 40 m, 60 m, and 80 m, a 30 m increase in air gap resulted in air velocity increases of 30.358, 17.495, 9.882, and 6.951, respectively. In Fig. 3b, the relationship between air gap and maximum air velocity shifts upward as caving scale increases, with velocity increments rising to 30.358, 37.811, 50.495, and 59.939, respectively. This is because larger caving scales result in greater kinetic energy, which intensifies the airblast. Furthermore, within the air gap range of 5–15 m, the curve exhibits the steepest slope, this is likely due to the shorter falling time of the rock, which reduces the influence of air resistance on the rock’s falling speed. As a result, the increase in kinetic energy is greater, while as the air gap continues to increase, air resistance has a larger impact on the falling speed, causing the kinetic energy increment to decrease. Therefore, the maximum air velocity in this range is more sensitive to changes in air gap. Thus, increasing muckpile height reduces the impact of air gap on airblast, while increasing caving scale enhances this impact. Additionally, airblast velocity is significantly more sensitive to air gap in the 5 m to 15 m range than in other ranges.

**Fig. 4:**
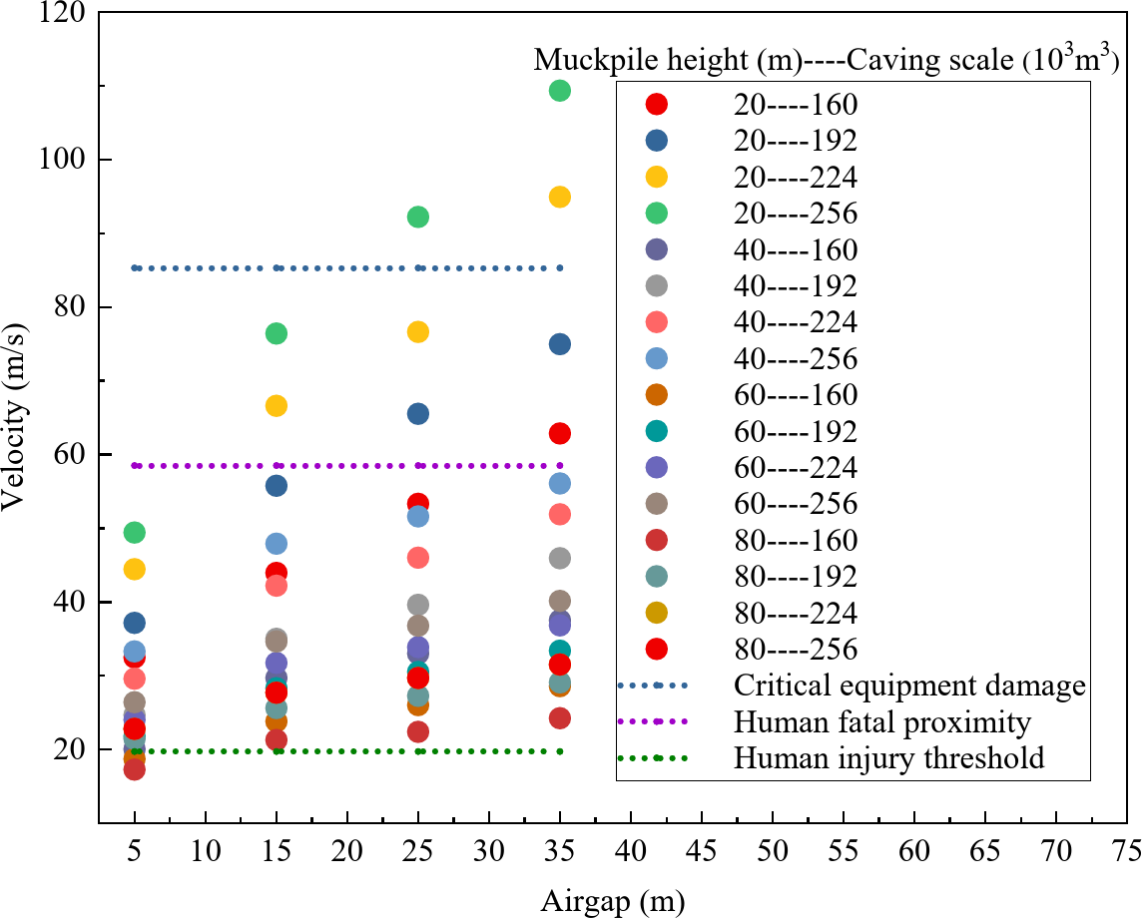
Distribution of maximum air velocity under different air gaps.

To determine the safety of different air gaps, this study summarized the distribution of maximum air velocity characteristic points under various combinations of factors. As shown in Fig. 4, only when the air gap is 5 m does the maximum air velocity remain below both the fatal threshold for humans and the critical value for equipment damage. Additionally, for air gaps greater than 5 m, the velocities exceed the injury threshold for humans, indicating that air gaps above 5 m are not suitable for regular construction under the combined factors in this study.

### Effect of muckpile height

**Fig. 5:**
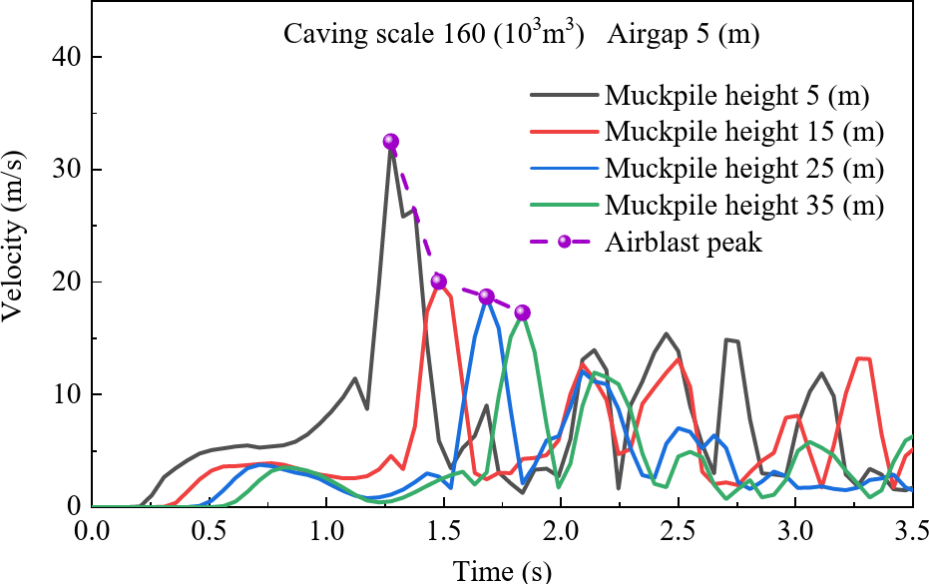
Air velocity evolution curves with different muckpile height.

To reveal the effect of muckpile height on airblast velocity, an analysis was conducted on airblast velocity distributions for four muckpile heights (20 m, 40 m, 60 m, and 80 m) with a 5 m air gap and a caving scale of 160 × 10^3^ m^3^. As shown in Fig. 5, with the increase in muckpile height, the overall trend of the airblast evolution curve remained unchanged, but the peak airblast velocity decreased nonlinearly. For muckpile heights of 20 m, 40 m, 60 m, and 80 m, the peak velocities were 32.479 m/s, 20.029 m/s, 18.688 m/s, and 17.269 m/s, respectively. This is because the muckpile acts as a buffer medium, a heighter muckpile provides a longer dissipation path for airblast energy while prolonging the obstruction time for compressed airflow. Consequently, a thicker muckpile enhances the attenuation effect on airblast, thereby reducing the peak intensity of the airblast. Additionally, the peak of the curve lags with increasing muckpile height, which is caused by the delayed arrival of the airblast at the drawpoint due to the increased muckpile height.

**Fig. 6:**
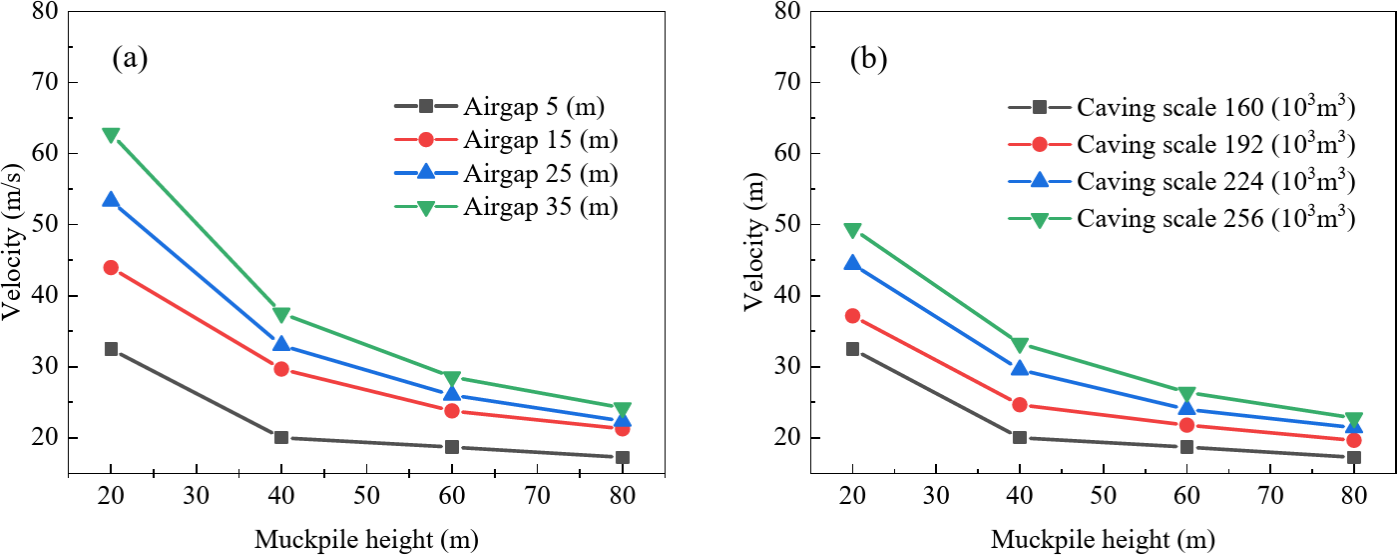
Relationship between muckpile height and maximum air velocity; (**a**) Effect of air gap on the relationship between muckpile height and maximum air velocity; (**b**) Effect of caving scale on the relationship between muckpile height and maximum air velocity.

To investigate whether the impact of muckpile height on airblast velocity is affected by air gap and caving scale, the effect of muckpile height on airblast velocity was analyzed based on four air gaps (5 m, 15 m, 25 m, and 35 m) and four caving scales (160 × 103 m^3^, 192 × 103 m^3^, 224 × 103 m^3^, and 256 × 103 m^3^). In Fig. 6a, the maximum air velocity at the drawpoint shows a negative correlation with muckpile height, with steeper curves observed for larger air gaps. When the air gap is 5 m, 15 m, 25 m, and 35 m, the maximum air velocity reductions due to muckpile height changes are 15.210 m/s, 22.667 m/s, 30.941 m/s, and 38.617 m/s, respectively. This is because increasing the air gap results in more released kinetic energy from falling ore, higher compressed air energy, and greater energy absorption by the muckpile, thereby enhancing the buffering effect on the airblast. Meanwhile, as shown in Fig. 6b, with increasing caving scale, the relationship curve between maximum air velocity and muckpile height shifts upward and becomes steeper. From the lowest to the highest caving scale, the variations in maximum air velocity caused by changes in muckpile height are 15.210 m/s, 17.494 m/s, 22.996 m/s, and 26.625 m/s, respectively. This is because larger-scale caving rock masses possess greater kinetic energy, resulting in increased energy absorption by the muckpile and, consequently, greater velocity variations. Additionally, in Fig. 6, the slope of the relationship curve between maximum air velocity and muckpile height decreases sharply with increasing muckpile height before leveling off. The slope is steepest between 20 m and 40 m, and flattens between 60 m and 80 m. This is because, in higher muckpile, most of the kinetic energy of the airblast has already been absorbed. As the muckpile height increases, the additional muckpile height have a progressively smaller effect on absorbing the airblast energy, thus reducing the attenuation effect. Therefore, an increase in air gap and caving scale enhances the airblast attenuation effect of muckpile height. However, as shown in Fig. 6a, b, the overall evolution curves of airblast still exhibit an upward trend with increasing air gap and caving scale. This suggests that the formation of airblast remains primarily governed by the kinetic energy of caving rock masses. Additionally, the attenuation effect of muckpile height on maximum air velocity diminishes as the muckpile thickness increases.

**Fig. 7:**
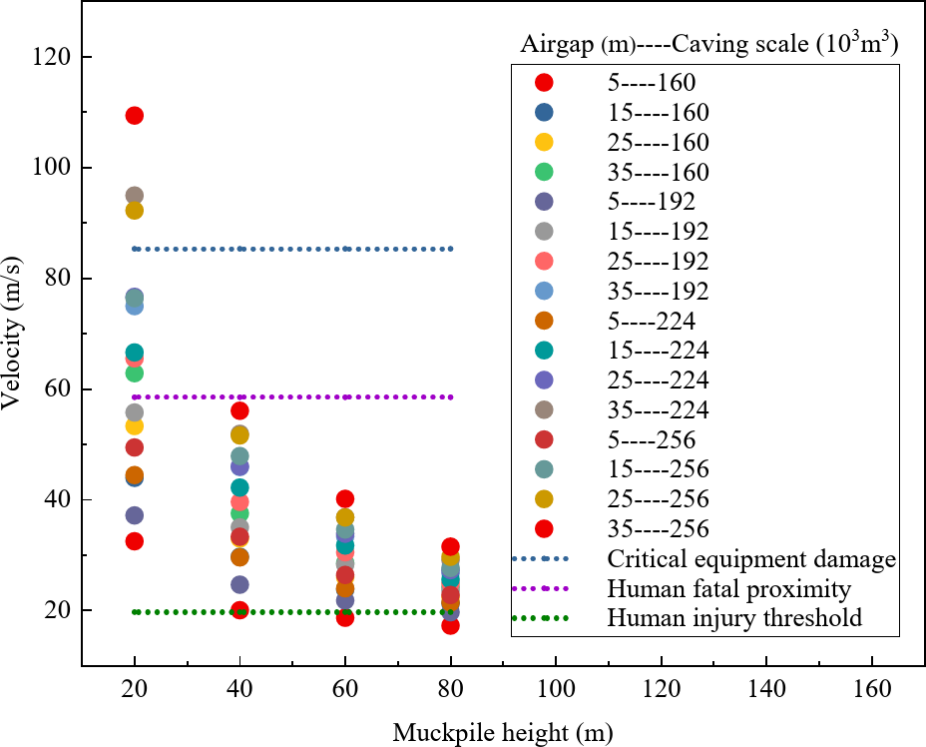
Distribution of maximum air velocity with different muckpile heights.

To investigate the safety of various muckpile heights, this study conducted a comprehensive analysis of the maximum air velocity characteristics across multiple factor combinations. As shown in Fig. 7, when the muckpile heights is 60–80 m, the maximum air velocity is below the lethal threshold for humans and the damage threshold for equipment, and in some areas, it is below the injury threshold for humans. However, in comparison, the overall air velocity is significantly lower when the muckpile heights is 80 m. Therefore, under the conditions of this study, muckpile heights of 60 m and 80 m show better safety performance than other thicknesses, with the 80 m height exhibiting the best safety.

### Effect of caving scale

**Fig. 8:**
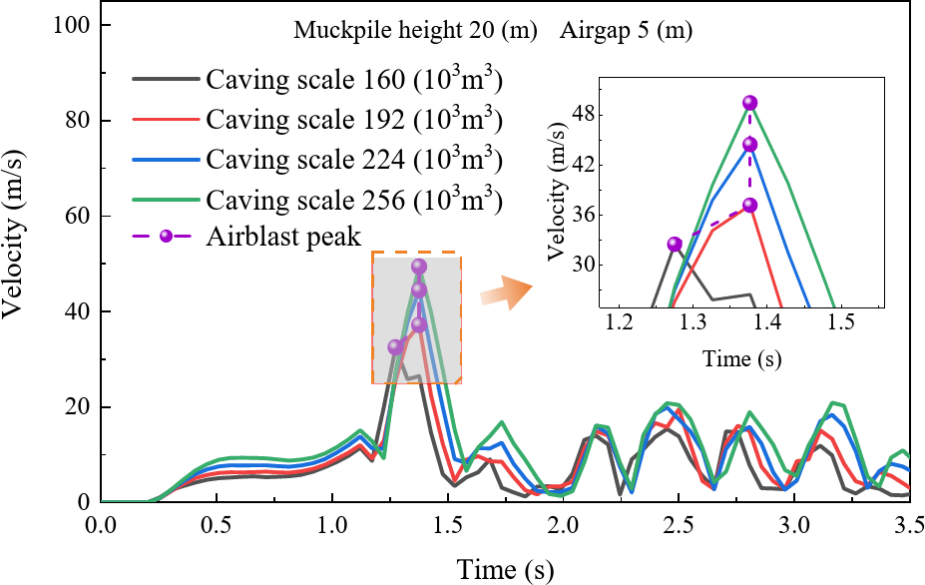
Air velocity evolution curves with different caving scales.

To investigate the effect of caving scale on airblast velocity, this study analyzed the airblast velocity distribution for caving scales of 160 × 10^3^ m^3^, 192 × 10^3^ m^3^, 224 × 10^3^ m^3^, and 256 × 10^3^ m^3^ with a muckpile heights of 20 m and an air gap of 5 m. As shown in Fig. 8, the evolution curve of the airblast moves upward with increasing caving scale, and the peak airblast velocity increases positively, with peak velocities of 32.479 m/s, 37.155 m/s, 44.436 m/s, and 49.422 m/s, respectively. This increase is attributed to the overall increase in the kinetic energy of the rock mass with a larger caving scale. Additionally, compared to other caving scales, the peak of the curve appears earlier for a caving scale of 160 × 10^3^ m^3^, which is due to the different target drawpoint chosen.

**Fig. 9:**
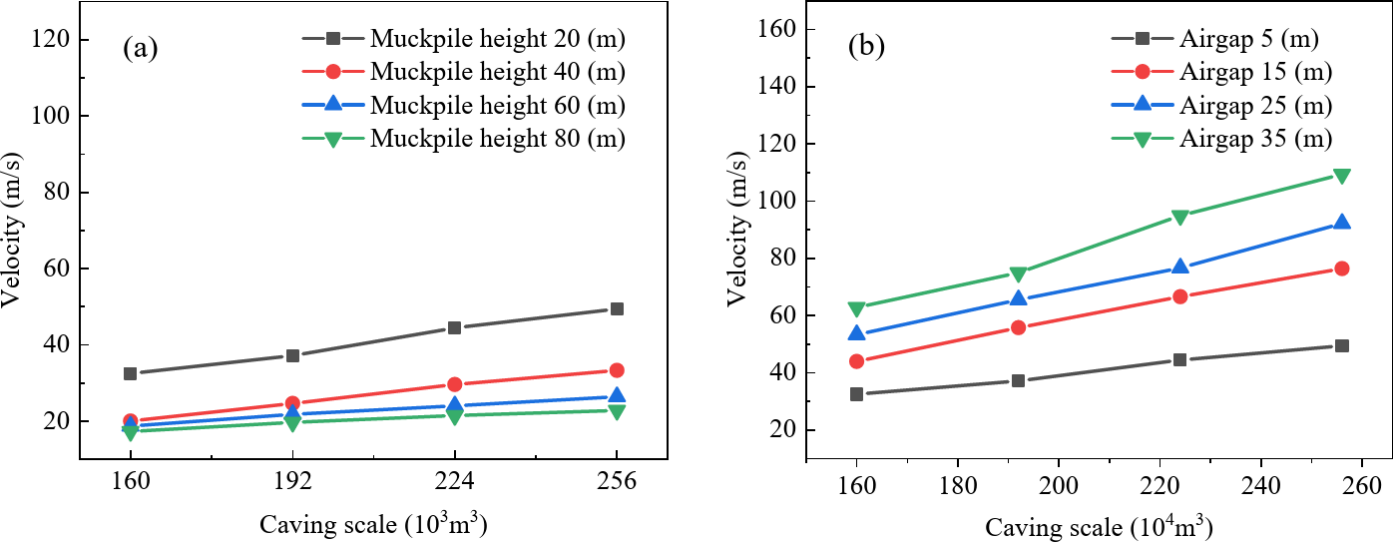
Relationship between caving scale and maximum air velocity; (**a**) Effect of muckpile height on the relationship between caving scale and maximum air velocity; (**b**) Effect of air gap on the relationship between caving scale and maximum air velocity.

To investigate whether the impact of caving scale on airblast is influenced by air gap and muckpile heights, this study analyzed the effects of caving scale on air velocity based on four air gaps (5 m, 15 m, 25 m, and 35 m) and four muckpile heights (20 m, 40 m, 60 m, and 80 m). As shown in Fig. 9, with increasing caving scale, the curve changes uniformly, approximating a linear relationship. In Fig. 9a, the maximum air velocity at drawpoint shows a positive correlation with caving scale, and the trend of curve change slightly slows with increasing muckpile heights. This is because increasing the muckpile height extends the energy dissipation path of the airblast while prolonging the obstruction time for compressed airflow, thereby reducing the amplification of air velocity. In Fig. 9b, the trend of curve change slightly increases with increasing air gap, as a larger air gap increases the falling distance of the rock mass, allowing it to gain higher kinetic energy during the caving process, thereby intensifying the airblast. Analysis indicates that there is an approximately linear relationship between caving scale and maximum air velocity. An increase in caving scale amplifies the effect of an increasing air gap on airblast enhancement. Conversely, a greater air gap further reinforces the impact of an increasing caving scale on airblast intensification.

**Fig. 10:**
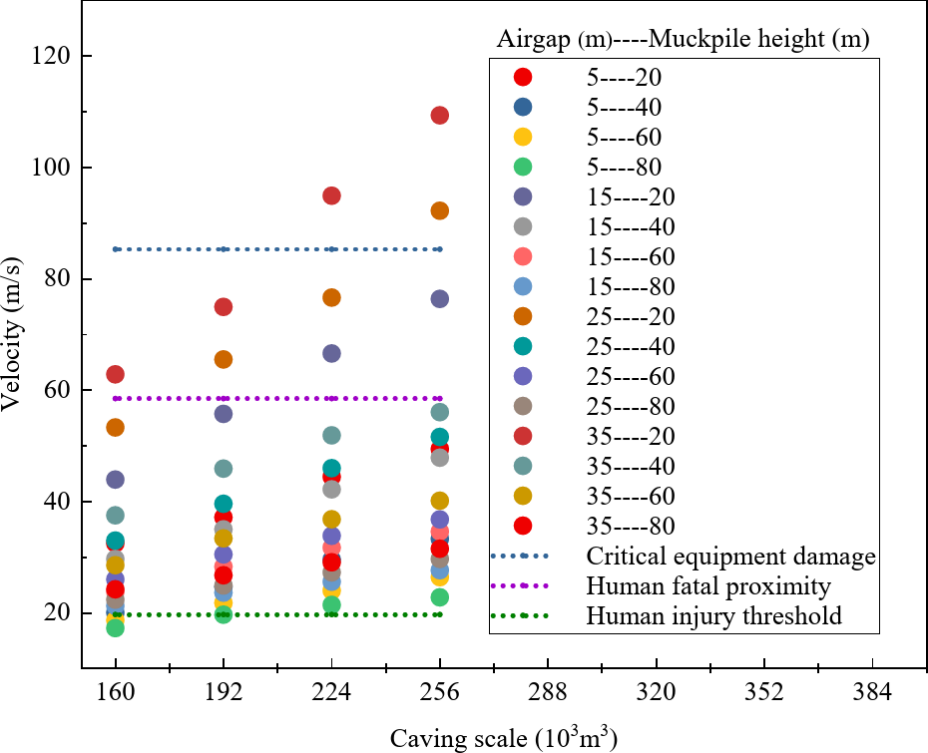
Distribution of maximum air velocity with different caving scales.

To assess the safety of different caving scales, this study comprehensively analyzed the distribution of maximum air velocity characteristics under various factor combinations. As shown in Fig. 10, only for a caving scale of 160 × 10^3^ m^3^ does the maximum air velocity remain well below the equipment damage threshold, and in some areas, the velocity is below the human injury threshold. Therefore, under the experimental conditions of this study, a caving scale of 160 × 10^3^ m^3^ demonstrates the best safety profile.

### Parameter sensitivity analysis

Sensitivity analysis is commonly characterized by a sensitivity coefficient^38^, which evaluates the response of indicators to uncertainty factors. The formula is as follows:

13 $${S}_{a}=\frac{\varDelta b}{\varDelta a}$$

where $\varDelta a$ represents the variation in parameter *a*, $\varDelta b$ represents the variation in the output variable, ${S}_{a}$ represents the sensitivity coefficient. The larger the coefficient, the more sensitive it is to parameter *a*.

To quantify the sensitivity of the caving scale, a unit of 10^3^ m^3^ was selected to represent the sensitivity to changes in caving scale at the 10^3^ m^3^ level. The results, as shown in Table 3, indicate that the average and maximum sensitivity coefficients for air gap and muckpile height are significantly higher than those for the caving scale. This indicates that, whether at a local or overall level, the maximum air velocity is more sensitive to air gap and muckpile height than to caving scale. This further demonstrates that the formation of airblast is primarily governed by the kinetic energy of caving rock masses. Moreover, the maximum and average sensitivity coefficients for air gap are slightly greater than those for muckpile height, with a difference of 0.033 in the maximum sensitivity coefficient and 0.043 in the average sensitivity coefficient. Therefore, in developing measures to prevent air blasts, for caving scale changes at the 10^3^ m^3^ level, the priority of the parameters should be air gap, muckpile height, and then caving scale.

**Table 3:** Sensitivity coefficients.

Parameter types	Parameter variation	Maximum air velocity	
		Maximum sensitivity coefficient	Average sensitivity coefficient
Air gap	10	2.698	0.710
Muckpile height	20	2.665	0.667
Caving scale	32	0.624	0.176

### Multivariate airblast model

Based on the Π-Buckingham theorem, a mathematical model was derived to establish the relationship between muckpile height, caving scale, air gap, and maximum air velocity. The model normalizes (rockfall velocity) with ${V}_{max}$ (maximum air velocity), and the equation as follows:

14 $$\frac{{V}_{max}}{{V}_{in}}=f({L}_{T},{V}_{R},{L}_{AG} )$$

where ${V}_{in}$ represents the rockfall velocity, which can be obtained from the following equation based on Newton’s laws of motion:

15 $${V}_{in}=\sqrt{2g{L}_{DB}}$$

After normalizing Muckpile height, Caving scale, and air gap, the following equation is obtained:

16 $$L\equiv \frac{{L}_{T}}{{D}_{DP}};\:\: V\equiv \frac{{V}_{R}}{{D}_{DP}^{3}};\:\: H\equiv \frac{{L}_{AG}}{{D}_{DP}}$$

The detailed expression of Eq. (13) is as follows:

17 $$\frac{{V}_{max}}{{V}_{in}}=\alpha{\left(\frac{{L}_{T}}{{D}_{DP}}\right)}^{\beta }{\left(\frac{{V}_{R}}{{D}_{DP}^{3}}\right)}^{\gamma }{\left(\frac{{L}_{AG}}{{D}_{DP}}\right)}^{\eta }$$

After simplification, Eq. (16) yields:

18 $$\frac{{V}_{\text{max }}}{{V}_{in}}=\alpha {L}^{\beta }{V}^{\gamma }{H}^{\eta }$$

After simplification, Eq. (17) yields:

19 $${V}_{max}=\sqrt{2g{L}_{DB}}{L}^{\beta }{V}^{\gamma }{H}^{\eta }$$

**Fig. 11:**
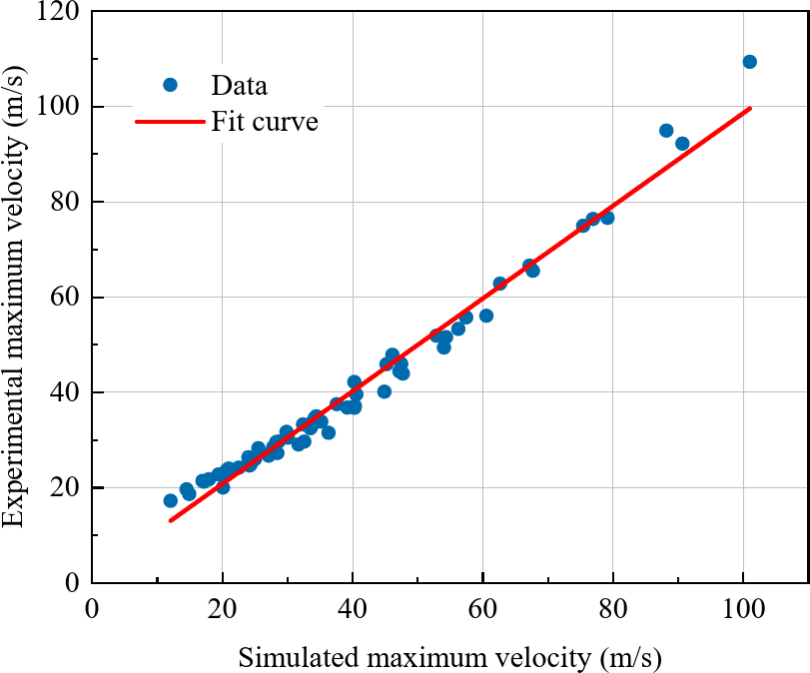
Comparison between simulated experiments and dimensionless multivariate model.

After calculation, in Eq. (18), $\alpha$ = 2.2677, $\beta$ = − 0.7380, $\gamma$ = 1.0156, $\eta$ = 0.3214. Comparing the maximum air velocity calculated from this model with the simulated experimental results, it can be seen from Fig. 11 that the deviation between the model values and experimental values is small. Therefore, through this model, the maximum air velocity can be determined using three parameters, providing important reference value for airblast prevention and related protective measures.

## Discussion

In practical engineering construction, to prevent the danger caused by airblast, it is necessary to control the potentially harmful air velocity within the acceptable range for equipment and personnel. Consequently, this study, combining the above analysis with actual engineering conditions, examines the safety reference values for air gap, muckpile height, and caving scale in practical engineering, aiming to provide scientific support for engineering practice and to ensure the safety of equipment and personnel.

According to the block caving mining technical regulations^34^, during production, the mine should control the draw to keep the air gap between the caving roof and the muckpile under 5 m. Nevertheless, based on the findings of this research, with a air gap greater than 5 m and a muckpile height of 40 m, the caving scale are less than 110.83, 94.31, and 84.80 × 10^3^ m^3^, respectively, and can be reduced to within safe limits, indicating that an air gap above 5 m is feasible during daily caving and ore drawing. However, since large-scale caving of the roof is difficult to predict and stabilize accurately^14^, this may lead to caving scale falling within an uncontrollable range, potentially causing severe airblast hazards. Additionally, when the air gap increases from 5 m to 15 m, the airblast velocity rises rapidly, and instability and danger also increase sharply. Therefore, in practical engineering, the higher the air gap, the greater the danger, and the more difficult it is to control. Under the current technological conditions, which are not sufficient to effectively control the high risks associated with a large air gap, choosing an air gap of 5 m or less is the safest and best option in actual construction.

The safest muckpile height is 80 m. However, while 80 m is clearly the safest muckpile height, considering that the airlast attenuation effect significantly weakens after the muckpile height increases to 40 m, and excessively thick muckpiles slow down the draw rate, thereby affecting overall production, the optimal muckpile height of 60 m is also suitable. Therefore, muckpile heights of 60 m and 80 m can serve as safety reference values. However, since these results are based on safety evaluations within a limited parameter range, practical engineering applications require safety designs or adjustments that consider the characteristics of the caved zone’s surrounding rock and environmental heterogeneity to ensure the effectiveness and safety of the design. The safety of an instantaneous caving scale of 160 × 10^3^ m^3^ is optimal. In actual production, attention should be paid to the development of large fault structures and fissures and the exposure and activation caused by undercutting.

## Conclusions

This paper, based on Mechsys, studied the impact of air gap, muckpile height, and caving scale on airblast, as well as the coupling effects between these parameters. Sensitivity analysis was used to compare the influence of each parameter on airblast. The results show that,

1. The air gap is positively correlated with airblast velocity, meaning that as the air gap increases, the kinetic energy of falling rock masses increases, resulting in a stronger airblast. However, further analysis indicates that increasing muckpile height mitigates the amplifying effect of the air gap on airblast, as a higher muckpile provides greater buffering capacity. Additionally, an increase in caving scale further enhances the influence of the air gap on airblast. A larger caving scale leads to greater total kinetic energy of falling rock masses, resulting in higher airblast energy accumulation.
2. Muckpile height is negatively correlated with airblast velocity, meaning that increasing muckpile height extends the energy dissipation path of the airblast and prolongs the obstruction time for compressed airflow, thereby reducing airblast intensity. An increase in air gap and caving scale enhances the airblast attenuation effect of the muckpile. This is because greater caving kinetic energy increases the energy of compressed air, leading to higher energy absorption by the muckpile and consequently improving its buffering effect on the airblast. However, despite the muckpile’s ability to weaken the airblast, the formation of airblast remains primarily governed by the kinetic energy of caving rock masses.
3. The caving scale is positively correlated with airblast velocity, meaning that an increase in the caving scale results in greater airblast intensity. However, increasing the muckpile height will reduce the effect of the caving scale on airblast, while increasing the air gap will enhance this effect.
4. Sensitivity analysis has clarified that, at a caving scale of 10^3^ m^3^, the air gap is the most critical parameter affecting airblast velocity,

Furthermore, based on Π-Buckingham theorem, this study presents a multivariate fitting model to describe the maximum airspeed at the ore pass during rock caving, with air gap, muckpile height, and caving scale as the fundamental parameters of the model. The study also discusses parameter design for practical engineering, showing that an air gap of 5 m or less is the safest parameter; muckpile heights of 60 m and 80 m are suitable choices in this study, but actual engineering design must consider the characteristics of the surrounding rock mass and environmental heterogeneity; caving scale design should be determined according to actual engineering needs, as there is no universally optimal value.

Based on the findings of this study, the primary measures for preventing airblast in mining operations should focus on controlling the air gap and caving scale. The study indicates that airblast formation is primarily governed by the kinetic energy of caving rock masses. Given the unpredictability of roof collapse, controlling the air gap is the most effective preventive measure. It is recommended to set the air gap at 5 m. For further optimization, the multiparameter coupling model described in section “Multivariate airblast model” can be used to adjust other ore drawing parameters, such as muckpile thickness and caving scale, ensuring that all parameters remain within a safe range to enhance operational safety.

## Data Availability

All data generated or analysed during this study are included in this published article.
